# Prevalence and antimicrobial resistance profile of pathogens isolated from patients with urine tract infections admitted to a university hospital in a medium-sized Brazilian city

**DOI:** 10.1590/S1678-9946202466003

**Published:** 2024-01-05

**Authors:** Mariana Negri, Bárbara Martins Lima, Renata dos Santos Batista Reis Woloszynek, Roberto Augusto Silva Molina, Carla Maria Ramos Germano, Débora Gusmão Melo, Leandro Cândido de Souza, Lucimar Retto da Silva de Avó

**Affiliations:** 1 Universidade Federal de São Carlos, Departamento de Medicina, São Carlos, São Paulo, Brazil; 2 Universidade Federal de São Carlos, Hospital Universitário, São Carlos, São Paulo, Brazil

**Keywords:** Infections, Urine, Multiple drug resistance, Drug therapy, Microbial sensitivity tests

## Abstract

This study aimed to determine the antibiotic profile of microorganisms isolated from urine samples of patients with community urine tract infections (UTI) admitted to the University Hospital of the Federal University of Sao Carlos to support an appropriate local empirical treatment. A retrospective cross-sectional study was conducted from October 2018 to October 2020. Data from 1,528 positive urine cultures for bacterial pathogens and antibiograms were tabulated. Bacterial species prevalence and their resistance profile were analyzed and compared by sex and age. For Gram-negative fermenting bacteria, resistance rates were compared between patients with previous hospitalization and the total of infections caused by this group. For comparisons, the Chi-square test was performed, using Fisher’s exact test when necessary (BioEstat program, adopting p ≤ 0.05). A multivariate analysis was applied to assess the effect of the studied variables in predicting multidrug resistance. Infections were more prevalent in women and older adults. Gram-negative bacteria represented 90.44% of total cultures. In both sexes, *E. coli* prevalence was significantly higher in adults compared with older adults (p < 0.0001). For several antibiotics, resistance rates were higher in the older adults compared with other ages and in patients with Gram-negative fermenting infections and previous hospitalization compared with the total of infections by this group of bacteria. The closer to the hospitalization, the higher the number of antibiotics with superior resistance rates. Resistance rates for aminoglycosides, carbapenems, ceftazidime, nitrofurantoin, piperacillin+tazobactam, and fosfomycin were less than 20%, considered adequate for empirical treatment. Only hospitalization in the previous 90 days was statistically significant in predicting infections by multidrug-resistant bacteria.

## INTRODUCTION

Urinary tract infections (UTIs) correspond to bacterial multiplication with tissue invasion of any part of the urinary tract^1^. Thus, they may affect the lower (urethritis, cystitis, prostatitis, and epididymitis) or upper tract (pyelonephritis and ureteritis) and have a broad spectrum of signs and symptoms^2^. Lower tract infections typically present as dysuria, urgency, increased voiding frequency with small urine volumes, nocturia, and suprapubic pain^3,4^. The upper infections are usually associated with nausea, vomiting, chills, fever, and pain in the flank and lower back, with or without lower tract symptoms^3^.

Enteric Gram-negative bacteria, especially *Escherichia coli*, are notably the most numerous etiologic agents of community-acquired UTIs^1^, which may be classified as uncomplicated or complicated. Even though this classification has changed over time, the most recent definitions confine uncomplicated UTI to lower or upper infections in non-pregnant women with no functional or anatomical abnormalities or comorbidities. All the other cases are considered complicated UTIs, i.e., male patients, pregnant women, individuals with relevant anatomical or functional abnormalities of the urinary tract, indwelling urinary catheters, renal diseases, and comorbidities^5^. In addition, community-acquired UTIs are those not associated to health care assistance, that is, they are infections present at the time of hospitalization^5^.

Undoubtedly, the gold standard for UTI diagnosis combines clinical and laboratorial data^2,6^. According to the current methods, once the presence of bacteria is confirmed and quantified, the sample is forwarded for urine culture and antibiogram procedures. The former enables species identification, whereas the latter examines antimicrobial susceptibility^7^. Such steps, however, require an incubation period of at least 24 h^8^. This prolonged period, combined with generally unpleasant symptomatology, contributes to the use of empirical treatment in the patient’s initial approach^9^. In this context, some specialists have recently reached the consensus that urine cultures are needed only under some specific circumstances, such as the suspicion of a resistant uropathogen, persistence of symptoms despite treatment, infection recurrence within four weeks, and the presence of atypical symptoms^6^.

The UTIs are the most prevalent urinary tract pathology, with a very high number of visits to the health care system, and they respond to a significant amount of empirical antimicrobial prescriptions^9,10^. To optimize therapy success as well as prevent the spread of bacterial resistance, empirical antibiotic therapy must be based on the most common local uropathogens and their antimicrobial susceptibility pattern^11^.

Considering this scenario, this study aimed to assess the frequency of uropathogens and their resistance profile. Additionally, for Gram-negative fermenting bacteria the resistance profile of patients was compared with previous hospitalization divided into groups according to the time interval between them. We also studied variables associated with multidrug-resistant bacteria infections. All these assessments enable more effective clinical management and a more rational and effective use of antibiotics.

## MATERIAL AND METHODS

### Study design and setting

We carried out an observational, descriptive, and retrospective cross-sectional study, which included positive urine cultures with antibiogram performed from October 1, 2018, to October 31, 2020, of patients admitted to the University Hospital of the Federal University of Sao Carlos (HU-UFSCar), Sao Paulo State, Brazil. Sao Carlos is a medium-sized city in the inland Sao Paulo State, 254 kilometers away from the state capital, with a population of 254,822 inhabitants and a human development index of 0.805 in 2010^12^. The HU-UFSCar is a teaching hospital that provides medium- and high-complexity services, with more than 20,000 annual visits, considering both urgent and emergency care and outpatient appointments.

This research was approved by the Human Research Ethics Committee at the Sao Carlos Federal University (process CAAE 3232492.0.0000.5504).

### Sampling and procedure

The uropathogen detection was performed in a clinical analysis laboratory by inoculating 0.1 mL of urine dilution in distilled water at 1:1,000 in chromogenic agar culture medium. After inoculation, the culture was stored in an incubator (35–37 °C) for 24 h or more, and the number of colonies was counted by multiplying the result obtained by 1,000 to obtain the number of colonies per milliliter. Thus, in this method, bacteria were quantified by evaluating the number of Colony Forming Units (CFU) per mL. The identification of the isolated species was made based on the morphology of the colonies, their coloration, and various biochemical tests. Bacteriuria was considered positive when ≥ 10^5^ CFU/ml were detected, and all the results were tabulated.

Only community-acquired infections were included in the analysis, thus, positive samples that met the criteria for healthcare-related urine infections – such as hospitalization within the last 48 h prior to the onset of UTI symptoms and/or to the urine collection – were excluded. Likewise, urine cultures representing duplicates (i.e., exams from the same patient) were also excluded from the study.

The antimicrobial susceptibility test was performed on urinary isolates using the disk diffusion method, according to BrCAST (Brazilian Committee on Antimicrobial Susceptibility Testing) and EUCAST (European Committee on Antimicrobial Susceptibility Testing) instructions. The following antimicrobials were tested: amikacin, amoxicillin+clavulanate, ampicillin, ampicillin+sulbactam, cephalothin, cefepime, cefotaxime, ceftazidime, ceftriaxone, cefuroxime, cefuroxime axetil, ciprofloxacin, ertapenem, fosfomycin, gentamicin, imipenem, levofloxacin, meropenem, nitrofurantoin, norfloxacin, piperacillin+tazobactam, sulfamethoxazole+trimethoprim, oxaxillin, penicillin, and vancomycin. Different drugs were tested for Gram-negative fermenting, Gram-negative non-fermenting, and Gram-positive species according to their spectrum of action. The low number of cases made it impossible to compare Gram-positive pathogens between sexes and age groups.

For each patient with positive urine culture, the following clinical data were collected: patient sex and age (three age groups were defined: “children” < 12 years-old, 12 ≤ “adults” < 65 years-old, and “older adults” ≥ 65 years-old), date of urine sampling, and uropathogen species. We also checked the retrospective medical records of each patient to identify hospitalizations within the maximum period of 90 days prior to the urine sampling. Previous studies have shown that exposure to antibiotics within three months was an independent predictor that characterized patients with multidrug-resistant pathogens, indicating broader-spectrum antibiotics as primary choice for treatment. Thus, patients who had previously been hospitalized were divided into three groups for comparison (previous hospitalization episodes in the last 30 days – excluding those with hospitalization within 48 h after hospital discharge, 31 to 60 days, and 61 to 90 days) to compare the pattern of resistance of the uropathogens and use the results to guide the rational use of empirical drugs, avoiding, when possible, the immediate introduction of broad-spectrum antibiotics, assuming that, the closer the hospitalization, the higher the multidrug resistance^13^.

### Descriptive and statistical analysis

Descriptive analyses were executed in Microsoft Excel 2010 (Microsoft, USA). Statistical analyses of the nonparametric results were performed by a Chi-squared test, using the Fisher’s exact test when samples were insufficient for a Chi-squared analysis. For such analyses, BioEstat 5.0 program (Informer Technologies, USA) was used, and a p-value of ≤ 0.05 was considered statistically significant. The resistance rates were calculated for Gram-negative (fermenting and non-fermenting) and Gram-positive species. Gram-negative species are the most prevalent species causing community UTI, thus their analysis is fundamental to provide bases for empirical treatment design.

A binomial logistic regression was performed using SPSS version 22.0 (IBM, USA) to ascertain the effects of age, gender, and hospitalization in the previous 90 days on the likelihood that participants have infections by multidrug-resistant bacteria – defined as pathogen non-susceptibility to at least one agent in three or more antimicrobial categories^14^.

## RESULTS

From October 2018-2020, HU-UFSCar recorded 1,528 positive urine cultures with bacterial uropathogens. A total of 72.38% of the infections were detected in women and 44.63% of all analyzed patients were aged 65 years old or more. Whereas the frequencies of children (18.61%) and older patients (52.98%) were significantly higher in men than in women (13.29% and 41.59%, respectively), the frequency of adults (45.12%) was significantly higher in women than in men (29.11%) (Table 1).

**Table 1:** Distribution by sex and age of the patients with positive urine cultures attending the University Hospital of the Federal University of Sao Carlos (Sao Paulo, Brazil) from October/2018 to October/2020.

Age (years)	Male		Female		Total	

	n	%	n	%	n	%
< 12	78	18.61	147	13.29	225	14.72
12-64	122	29.11	499	45.12	621	40.05
≥ 65	222	52.98	460	41.59	682	44.63
Total	419	100	1106	100	1528	100

Gram-negative bacilli represented 1,382 (90.44%) of all detected bacteria, and the remaining 146 isolates (9.56%) were Gram-positive cocci. Among all bacilli, 1,304 (94.79%) were species of the order Enterobacterales (fermenting species), and 78 (5.21%) were non-fermenting species. Among the five most prevalent species, there were four bacilli (*Escherichia coli* – 63.87%; *Klebsiella pneumoniae* – 10.34%; *Proteus mirabilis* – 5.69%; and *Pseudomonas aeruginosa* – 4.06%), and one coccus (*Enterococcus faecalis* – 4.06%). Comparing both sexes, these five most frequent species in the analyses considering both sexes altogether were also the most common species for each sex separately (Table 2).

**Table 2:** Frequency by sex and age of bacteria isolated from patients with positive urine cultures attending the University Hospital of the Federal University of Sao Carlos (Sao Paulo, Brazil) from October/2018 to October/2020.

Gram-negative bacteria								

	Male				Female			

	< 12, n (%)	12-64, n (%)	≥ 65, n (%)	Total, n (%)	< 12, n (%)	12-64, n (%)	≥ 65, n (%)	Total, n (%)
**GRAM-NEGATIVE FERMENTING SPECIES:**								
Citrobacter braakii	0	0	1 (0.5)	1 (0.3)	0	1 (0.2)	0	1 (0.1)
Citrobacter koseri	0	1 (0.9)	5 (2.6)	6 (1.6)	1 (0.7)	3 (0.7)	2 (0.5)	6 (0.6)
Citrobacter freundii	0	0	0	0	0	1 (0.2)	1 (0.2)	2 (0.2)
Enterobacter aerogenes	0	1 (0.9)	3 (1.5)	4 (1.1)	0	3 (0.7)	1 (0.2)	4 (0.4)
Enterobacter cloacae	1 (1.4)	2 (1.8)	2 (1.0)	5 (1.3)	0	3 (0.7)	4 (1.0)	7 (0.7)
Escherichia coli	38 (52.8)	69 (63.3)^**b**^	97 (50.0)^**b**^	204 (54.4)^**a**^	114 (83.8)^**b**^	384 (84.0)^**b**^	274 (66.2)^**b**^	772 (76.7)^**a**^
Klebsiella oxytoca	1 (1.4)	4 (3.7)	1 (0.5)	6 (1.6)	3 (2.2)	2 (0.4)	5 (1.2)	10 (1.0)
Klebsiella pneumoniae	8 (11.1)	16 (14.7)	36 (18.6)	60 (16.0)^**a**^	5 (3.7)^**b**^	35 (7.7)^**b**^	58 (14.0)^**b**^	98 (9.7)^**a**^
Morganella morganii	2 (2.8)	2 (1.8)	5 (2.6)	9 (2.4)^**a**^	0	0	8 (1.9)	8 (0.8)^**a**^
Proteus mirabilis	17 (23.6)^**b**^	8 (7.3)^**b**^	10 (5.2)^**b**^	35 (9.3)^**a**^	9 (6.6)	19 (4.2)	24 (5.8)	52 (5.2)^**a**^
Proteus penneri	0	1 (0.9)	2 (1.0)	3 (0.8)	0	0	0	0
Proteus vulgaris	0	0	0	0	1 (0.7)	0	0	1 (0.1)
Providencia rettgeri	0	2 (1.8)	0	2 (0.5)	0	0	1 (0.2)	1 (0.1)
Providencia stuartii	0	0	0	0	0	0	2 (0.5)	2 (0.2)
Providencia sp	1 (1.4)	0	0	1 (0.3)	0	0	0	0
Raoultella ornithinolytica	0	0	0	0	0	1 (0.2)	3 (0.7)	4 (0.4)
Raoultella planticola	0	0	1 (0.5)	1 (0.3)	0	0	0	0
Serratia marcescens	0	1 (0.9)	2 (1.0)	3 (0.8)	1 (0.7)	1 (0.2)	0	2 (0.2)
**GRAM-NEGATIVE NON-FERMENTING SPECIES:**								
Acinetobacter baumannii complex	0	0	4 (2.1)	4 (1.1)	0	1 (0.2)	1 (0.2)	2 (0.2)
Pseudomonas aeruginosa	3 (4.2)	2 (1.8)^**b**^	24 (12.4)^**b**^	29 (7.7)^**a**^	2 (1.5)^**b**^	3 (0.7)^**b**^	28 (6.8)^**b**^	33 (3.3)^**a**^
Pseudomonas putida	0	0	0	0	0	0	1 (0.2)	1 (0.1)
Burkholderia cepacia	1 (1.4)	0	1 (0.5)	2 (0.5)	0	0	1 (0.2)	1 (0.1)
**Total**	72 (100)	109 (100)	194 (100)	375 (100)	136 (100)	457 (100)	414 (100)	1007 (100)
**GRAM-POSITIVE SPECIES:**								
Enterococcus faecalis	4 (66.7)	3 (23.1)^**b**^	19 (67.9)^**b**^	26 (55.3)^**a**^	5 (45.4)	6 (14.3)^**b**^	25 (54.3)^**b**^	36 (36.4)^**a**^
Enterococcus faecium	0	0	0	0	0	0	3 (6.5)	3 (3.0)
Staphylococcus aureus	1 (16.7)	5 (38.5)	4 (14.3)	10 (21.3)	0	7 (16.7)	5 (10.9)	12 (12.1)
Staphylococcus capitis	0	1 (7.7)	0	1 (2.1)	0	0	0	0
Staphylococcus epidermidis	1 (16.7)	2 (15.4)	3 (10.7)	6 (12.8)	0	0	3 (6.5)	3 (3.0)
Staphylococcus haemolyticus	0	1 (7.7)	0	1 (2.1)	0	5 (11.9)	0	5 (5.1)
Staphylococcus hominis subsp. hominis	0	1 (7.7)	1 (3.6)	2 (4.3)	1 (9.1)	1 (2.4)	2 (4.3)	4 (4.0)
Staphylococcus saprophyticus	0	0	0	0	2 (18.2)	13 (30.9)^**b**^	1 (2.2)^**b**^	16 (16.2)^**a**^
Staphylococcus warneri	0	0	0	0	1 (9.1)	4 (9.5)	0	5 (5.1)
Streptococcus agalactiae **(Group B)**	0	0	1 (3.6)	1 (2.1)^**a**^	2 (18.2)	5 (11.9)	7 (15.2)	14 (14.1)^**a**^
Streptococcus gallolyticus	0	0	0	0	0	1 (2.4)	0	1 (1.0)
**Total**	6 (100)	13 (100)	28 (100)	47 (100)	11 (100)	42 (100)	46 (100)	99 (100)

^a^significant difference in pathogen frequencies between males and females; ^b^significant difference in pathogen frequencies between the indicated age groups. Significance level p ≤ 0.05.

Regarding the comparison of Gram-negative species between sexes, only *E. coli* showed a significantly higher frequency in women than in men. In contrast, *Morganella morganii, K. pneumoniae, P. mirabilis,* and *P. aeruginosa* had a superior frequency in men. In the comparison of Gram-negative species between age groups, for men, the frequency of *E. coli* was significantly higher in adults than in older patients; the frequency of *P. mirabilis* was significantly higher in children than in adults and older patients; and the frequency of *P. aeruginosa* was significantly higher in the older adults than in adults. For women, the frequency of *E. coli* in older patients was lower than those of the other age groups, whereas the frequency of *K. pneumoniae* and *P. aeruginosa* in older patients was higher than in the other age groups (Table 2).

The Gram-positive species were also more frequent in women (67.81%) than in men (32.19%). For both sexes, *E. faecalis* was the most numerous Gram-positive species. *Staphylococcus aureus* and *Staphylococcus epidermidis* were the second and third most frequent in men, respectively. *Staphylococcus saprophyticus* and *Streptococcus agalactiae* (Group B) were the second and third most frequent in women. *S. saprophyticus* was only detected in female patients, as were *Enterococcus faecium, Staphylococcus warneri*, and *Streptococcus gallolyticus*. The comparison between age groups showed significantly higher rates in older adults compared with adults for *E. faecalis* in both sexes. For women, *S. saprophyticus* was more frequent in adults compared with the older patients (Table 2).

Tables 3 and 4 show the antimicrobial resistance rates of Gram-negative and Gram-positive species, respectively. For many antibiotics, the comparison of resistance rates of Gram-negative fermenting species between sexes revealed that men had higher resistance rates than women. For both sexes, such rates were higher in older patients as well (Table 5). No significant difference was found between sexes and age groups of Gram-negative non-fermenting species.

**Table 3:** Antibiotic resistance pattern of Gram-negative bacteria isolated from urine samples of patients attending the University Hospital of the Federal University of Sao Carlos (Sao Paulo, Brazil) from October/2018 to October/2020.

Gram-negative bacteria						

Antibiotics	Total		Fermenting species		Non-fermenting species	

	N	n (%)	N	n (%)	N	n (%)
Amikacin	1372	42 (3.1)	1304	32 (2.5)	68	10 (14.7)
Gentamicin	1371	211 (15.4)	1303	195 (15.0)	68	16 (23.5)
Cephalothin	275	155 (56.4)	275	155 (56.4)	-	-
Cefuroxime	1288	470 (36.5)	1287	469 (36.4)	1	1 (100)
Cefuroxime axetil	918	410 (44.7)	917	409 (44.6)	1	1 (100)
Ceftazidime	639	127 (19.9)	610	120 (19.7)	29	7 (24.1)
Ceftriaxone **/** Cefotaxime	1308	391 (29.9)	1306	389 (29.8)	2	2 (100)
Cefepime	1367	357 (26.1)	1303	344 (26.4)	64	13 (20.3)
Ertapenem	1307	76 (5.8)	1306	76 (5.8)	1	0
Imipenem	631	69 (10.9)	605	64 (10.6)	26	5 (19.2)
Meropenem	1375	50 (3.6)	1304	36 (2.8)	71	14 (19.7)
Ciprofloxacin	1374	439 (32.0)	1309	420 (32.1)	65	19 (29.2)
Levofloxacin	70	21 (30.0)	66	20 (30.3)	4	1 (25.0)
Norfloxacin	811	328 (40.4)	794	322 (40.6)	17	6 (35.3)
Sulfamethoxazole+trimethoprim	815	300 (36.8)	801	288 (36.0)	14	12 (85.7)
Nitrofurantoin	470	94 (20.0)	470	94 (20.0)	-	-
Amoxicillin+clavulanate	782	200 (25.6)	782	200 (25.6)	-	-
Ampicillin	1308	905 (69.2)	1307	904 (69.2)	1	1 (100)
Ampicillin+sulbactam	611	310 (50.7)	609	308 (50.6)	2	1 (100)
Piperacillin+tazobactam	1352	242 (17.9)	1289	224 (17.4)	63	18 (28.6)
Fosfomycin	62	1 (1.6)	62	1 (1.6)	-	-

N = number of isolates tested for the respective antimicrobial; n (%) = absolute number (percentage) of isolates among those tested that showed resistance to the antibiotic; - = not tested. Significance level p ≤ 0.05.

**Table 4:** Antibiotic resistance pattern of Gram-positive bacteria isolated from urine samples of patients attending the University Hospital of the Federal University of Sao Carlos (Sao Paulo, Brazil) from October/2018 to October/2020.

Gram-positive bacteria										

	Enterococcus spp.		Total Staphylococcus		Coagulase-positive staphylococci		Coagulase-negative staphylococci		Streptococcus spp.	

	N	n(%)	N	n(%)	N	n(%)	N	n(%)	N	n(%)
Amikacin	1	0	-	-	0	-	0	-	0	-
Gentamicin	-	-	-	-	0	-	0	-	0	-
Ceftriaxone / Cefotaxime	1	0	-	-	0	-	0	-	1	0
Cefepime	-	-	-	-	0	-	0	-	1	0
Ciprofloxacin	17	7 (41.2)	13	2 (15.4)	2	1 (50.0)	11	1 (9.1)	1	0
Levofloxacin	47	14 (29.8)	60	17 (28.3)	22	10 (45.5)	38	7 (18.4)	15	3 (20.0)
Norfloxacin	9	3 (33.3)	5	0	-	-	5	0	1	1 (100)
Sulfamethoxazole+trimethoprim	-	-	65	3 (4.6)	22	0.0	43	3 (7.0)	3	0
Nitrofurantoin	45	3 (6.7)	-	-	-	-	-	-	-	-
Amoxicillin+clavulanate	-	-	6	4 (66.7)	2	2 (100)	4	2 (50.0)	-	-
Ampicillin	62	1 (1.6)	6	100	2	2 (100)	4	4 (100)	10	0
Ampicillin+sulbactam	-	-	6	4 (66.7)	2	2 (100)	4	2 (50.0)	-	-
Oxacillin	1	1 (100)	63	26 (41.3)	22	9 (40.9)	41	17 (41.5)	-	-
Penicillin	-	-	51	44 (86.3)	22	17 (77.3)	29	27 (93.1)	4	2 (25.0)
Vancomycin	65	1 (1.5)	65	0	24	0	43	0	16	0

N = number of isolates tested for the respective antimicrobial; n (%) = absolute number (percentage) of isolates among those tested that showed resistance to the antibiotic; - = not tested. Significance level p ≤ 0.05.

**Table 5:** Antibiotic resistance pattern by sex and age of Gram-negative fermenting bacteria isolated from urine samples of patients attending the University Hospital of the Federal University of Sao Carlos (Sao Paulo, Brazil) from October/2018 to October/2020.

Gram-negative fermenting bacteria																

Antibiotics	Male								Female							

	< 12 years		12-64 years		≥ 65 years		Total		< 12 years		12-64 years		≥ 65 years		Total	

	N	n (%)	N	n (%)	N	n (%)	N	n (%)	N	n (%)	N	n (%)	N	n (%)	N	n (%)
Amikacin	68	1 (1.5)	106	2 (1.9)	165	6 (3.6)	339	9 (2.7)	131	0	452	9 (2.0)	382	14 (3.7)	965	23 (2.4)
Gentamicin	68	5 (7.4)^**b**^	106	23 (21.7)^**b**^	165	37 (22.4)^**b**^	339	65 (19.2)^**a**^	131	11 (8.4)^**b**^	452	50 (11.1)^**b**^	381	69 (18.1)^**b**^	964	130 (13.5)^**a**^
Cephalothin	14	6 (42.9)	17	13 (76.5)	38	23 (60.5)	69	42 (60.9)	30	13 (43.3)	91	55 (60.4)	85	45 (52.9)	206	113 (54.9)
Cefuroxime	67	18 (26.9)^**b**^	100	41 (41.0)^**b**^	158	88 (55.7)^**b**^	325	147 (45.2)^**a**^	133	27 (20.3)^**b**^	451	146 (32.4)^**b**^	378	149 (39.4)^**b**^	962	322 (33.5)^**a**^
Cefuroxime axetil	50	17 (34.0)^**b**^	68	33 (48.5)	108	67 (62.0)^**b**^	226	117 (51.8)^**a**^	100	28 (28.0)^**b**^	322	129 (40.1)^**b**^	269	135 (50.2)^**b**^	691	292 (42.3)^**a**^
Ceftazidime	35	5 (14.3)^**b**^	45	10 (22.2)^**b**^	81	34 (42.0)^**b**^	161	49 (30.4)^**a**^	63	4 (6.3)^**b**^	215	30 (14.0)	171	37 (21.6)^**b**^	449	71 (15.8)^**a**^
Ceftriaxone **/** Cefotaxime	68	15 (22.1)^**d**^	106	33 (31.1)^**b**^	164	76 (46.3)^**b**^	338	124 (36.7)^**a**^	133	21 (15.8)^**b**^	453	127 (28.0)^**b**^	382	117 (30.6)^**b**^	968	265 (27.4)^**a**^
Cefepime	68	12 (17.6)^**b**^	107	31 (29.0)	164	67 (40.9)^**b**^	339	110 (32.4)^**a**^	132	14 (10.6)^**b**^	452	110 (24.3)^**b**^	380	110 (28.9)^**b**^	964	234 (24.3)^**a**^
Ertapenem	68	1 (1.5)^**b**^	107	6 (5.6)	165	19 (11.5)^**d**^	340	26 (7.6)	133	4 (3.0)	451	22 (4.9)	382	24 (6.3)	966	50 (5.2)
Imipenem	34	7 (20.6)	45	6 (13.3)	80	19 (23.8)	159	32 (20.1)^**a**^	62	2 (3.2)	214	11 (5.1)^**b**^	170	19 (11.2)^**b**^	446	32 (7.2)^**a**^
Meropenem	68	0^**b**^	106	2 (1.9)	165	13 (7.9)^**b**^	339	15 (4.4)^**a**^	132	2 (1.5)	451	6 (1.3)	382	13 (3.4)	965	21 (2.2)^**a**^
Ciprofloxacin	68	9 (13.2)^**b**^	107	45 (42.1)^**b**^	165	86 (52.1)^**b**^	340	140 (41.2)^**a**^	133	16 (12.0)^**b**^	453	102 (22.5)^**b**^	383	162 (42.3)^**b**^	969	280 (28.9)^**a**^
Levofloxacin	4	0^**b**^	3	2 (66.7)	11	9 (81.8)^**b**^	18	11 (61.1)^**a**^	5	0	25	4 (16.0)	18	5 (27.8)	48	9 (18.8)^**a**^
Norfloxacin	38	11 (28.9)^**b**^	67	32 (47.8)	99	60 (60.6)^**d**^	204	103 (50.5)^**a**^	79	16 (20.3)^**b**^	268	87 (32.5)^**b**^	243	116 (47.7)^**b**^	590	219 (37.1)^**a**^
Sulfamethoxazole+ trimethoprim	38	13 (34.2)	68	22 (32.4)	101	49 (48.5)	207	84 (40.6)	81	32 (39.5)	270	80 (29.6)	243	92 (37.9)	594	204 (34.3)
Nitrofurantoin	23	7 (30.4)	30	4 (13.3)	57	13 (22.8)	110	24 (21.8)	56	6 (10.7)	162	32 (19.8)	142	32 (22.5)	360	70 (19.4)
Amoxicillin+ clavulanate	37	8 (21.6)	65	19 (29.2)	100	39 (39.0)	202	66 (32.7)^**a**^	78	12 (15.4)^**b**^	263	55 (20.9)	239	67 (28.0)^**b**^	580	134 (23.1)^**a**^
Ampicillin	68	42 (61.8)^**b**^	107	83 (77.6)^**b**^	165	129 (78.2)^**b**^	340	254 (74.7)^**a**^	133	86 (64.7)	451	293 (65.0)	383	271 (70.8)	967	650 (67.2)^**a**^
Ampicillin+ Sulbactam	35	15 (42.9)	45	28 (62.2)	79	48 (60.8)	159	91 (57.2)	63	30 (47.6)	217	102 (47.0)	170	85 (50.0)	450	217 (48.2)
Piperacillin+ Tazobactam	68	10 (14.7)^**b**^	103	19 (18.4)	164	46 (28.0)^**b**^	335	75 (22.4)^**a**^	133	14 (10.5)^**d**^	446	66 (14.8)	375	69 (18.4)^**b**^	954	149 (15.6)^**a**^
Fosfomycin	2	0	3	0	8	0	13	0	8	0	21	0	20	1 (5.0)	49	1 (2.0)

N = number of isolates tested for the respective antimicrobial; n (%) = absolute number (percentage) of isolates among those tested that shown resistance to the antibiotic; ^a^significant difference in pathogen frequencies between males and females; ^b^significant difference in antimicrobial resistance between the indicated age groups. Significance level p ≤ 0.05.

Due to the limited sample size, only Gram-negative fermenting species were included in the analysis of patients with previous hospitalization episodes in the last 90 days (n = 165). No significant differences were found between the resistance rates among all defined groups for all tested antibiotics (previous hospitalization in the last 30 days, 31 to 60 days, and 61 to 90 days). However, when compared with the total rates calculated based on all 1,310 Gram-negative fermenting species, uropathogens isolated from patients who had previously been hospitalized showed higher resistance rates for many antibiotics. The closer the hospitalization, the higher the number of antibiotics with superior resistance rates (Table 6).

**Table 6:** Antibiotic resistance pattern of Gram-negative fermenting species isolated from patients with previous periods of hospitalization in the last 30 days, 31-60 days, and 61-90 days.

Gram-negative fermenting species in patients with previous hospitalization						

Antibiotics	30 days		31-60 days		61-90 days	

	N	n (%)	N	n (%)	N	n (%)
Amikacin	48	4 (8.3)	29	2 (6.9)	20	0
Gentamicin	48	11 (22.9)	29	7 (24.1)	20	3 (15.0)
Cephalothin	8	8 (100)*	3	2 (66.7)	3	0
Cefuroxime	47	30 (63.8)*	29	17 (58.6)*	20	11 (55.0)
Cefuroxime axetil	30	20 (66.7)*	19	10 (52.6)	16	12 (75.0)*
Ceftazidime	17	7 (41.2)	11	4 (36.4)	10	4 (40.0)
Ceftriaxone **/** Cefotaxime	48	27 (56.2)*	29	15 (51.7)*	20	7 (35.0)
Cefepime	48	25(52.1)*	29	14 (48.3)*	19	6 (31.6)
Ertapenem	48	5 (10.4)	29	5 (17.2)*	20	2 (10.0)
Imipenem	17	2 (11.8)	11	3 (27.3)	10	2 (20.0)
Meropenem	48	40 (8.3)	29	5 (17.2)*	20	2 (10.0)
Ciprofloxacin	48	30 (62.5)*	29	14 (48.3)	20	9 (45.0)
Levofloxacin	3	3 (100)	-	-	2	0
Norfloxacin	34	23 (67.6)*	19	11 (57.9)	12	4 (33.3)
Sulfamethoxazole+trimethoprim	34	18 (52.9)	20	8 (40.0)	12	6 (50.0)
Nitrofurantoin	18	4 (22.2)	4	1 (25.0)	6	1 (16.7)
Amoxicillin+clavulanate	32	15 (46.9)*	20	9 (45.0)	13	6 (46.1)
Ampicillin	48	43 (89.6)*	29	25 (86.2)	20	15 (75.0)
Ampicillin+sulbactam	17	12 (70.6)	11	6 (54.5)	9	3 (33.3)
Piperacillin+tazobactam	47	18 (38.3)*	29	7 (34.5)*	20	6 (30.0)
Fosfomycin	2	0	-	-	-	-

N = number of isolates tested for the respective antimicrobial; n (%) = absolute number (percentage) of isolates among those tested that shown resistance to the antibiotic; *significant difference in antimicrobial resistance between the group with previous hospitalization and the total antimicrobial resistance of Gram-negative fermenting species; - = not tested. Significance level p ≤ 0.05.

The logistic regression model was not statistically significant, χ^2^(3) = 5.486, p = 0.139. The model explained less than 1% (Nagelkerke R2) of the variance in multidrug-resistant bacteria and did not increase the number of correctly classified cases. Of the three predictor variables, only previous hospitalization in the last 90 days was statistically significant. Patients with previous hospitalization had 1.574 times higher odds of exhibiting multidrug-resistant bacteria.

## DISCUSSION

Apart from providing relevant information on the local epidemiological aspects of urinary tract infections (UTIs), this study also reinforced some general knowledge on this pathology. First, the proportionality of UTI between sexes – with deviation towards women – once again corroborated a well-documented proportion. Indeed, 50–80% of women will experience at least one episode of UTI in their lifetime^1^. Females are predisposed to UTI for many reasons, including anatomical characteristics (shorter urethra and its proximity to vagina and anus), the use of certain spermicides, pregnancy (with dilation of the ureter and renal pelvis facilitating reflux), the sexual act itself (with correlation between the frequency of sexual intercourses and the chance of acquiring a UTI), poor hygiene, menopause, and diabetes^1,15,16^.

The observed differences in the prevalence rates of each age group between both sexes deserve some consideration. A higher occurrence of UTI in female adults compared with this same age group in males might be related to the aspects previously mentioned, and such a high number of adult women might push down the percentages of children and older patients with UTI in females. Furthermore, previous research has shown that male infants have a slightly higher frequency of UTIs. This proportion would be related to a greater occurrence of congenital anomalies in the urinary tract in individuals of this sex^1^, with *P. mirabilis* as the main etiological agent^17^. Herein, we observed a higher percentage of *P. mirabilis* in male patients and, among them, in individuals younger than 12 years old.

Besides that, after the fifth decade of life, the incidence of UTI in males increases^1^. In our study, men made up a larger proportion of older patients than women. The high occurrence in older males might be explained by alterations such as prostatic hypertrophy, which provokes urinary flow obstruction and incomplete bladder emptying^1,18^. In older women, the occurrence of UTIs is facilitated by anatomic-functional changes associated with menopause, such as genitourinary prolapse, changes in pH and local flora, decreased bladder capacity, and increased urinary incontinence^18,19^. In the older group in general, immunosenescence, immobility, increased contamination with feces due to fecal incontinence, and the presence of systemic diseases are factors that favor the development of UTIs^1,20^.

The order Enterobacterales, especially *E. coli*, has been widely implicated as the main etiological agent of UTI. In the studied locality, *E. coli* frequency was followed by *K. pneumoniae* and *P. mirabilis*. The Gram-negative non-fermenting species *P. aeruginosa* and the Gram-positive *E. faecalis* were the fourth and fifth most common species, respectively. These same agents were observed in several other studies, with some local variations^21-28^. This same ranking was seen within each sex group, separately. However, *E. coli* was significantly more frequent in females, whereas the other species, except *E. faecalis*, were significantly more frequent in males.

Although *E. coli* was the most prevalent pathogen in all age groups, the frequency of this species seems to decrease in older patients, followed by an increase in the rates of other uropathogens in this age group. On the one hand, *E. coli* was less prevalent in the older patients than in male adults, whereas this species was less prevalent in older patients than among female adults and children. On the other hand, *K. pneumoniae* was more frequent in older patients compared with the other age groups in females; *P. aeruginosa* was more frequent in older patients compared with male adults and more frequent in older patients compared with the other age groups in women; and *E. faecalis* was more frequent in older patients than in adults of both sexes. Other studies had already observed this proportionality shift^20^ that might be related to the increase in complicated UTIs in older patients^3,19^.

Some studies cite the species *Staphylococcus saprophyticus* as one of the most frequent etiologic agents of community-acquired UTI^1,29^. In spite of that, this species was not among the most numerous in our global analysis. The UTIs caused by *S. saprophyticus* are related to sexual activity and tend to affect women of reproductive age. In line with such aspects, this species was only detected in women, with adults having the highest rate.

To design an empirical treatment, it is important to consider the resistance pattern of Gram-negative fermenting bacteria, since this group is responsible for most UTIs. Resistance rates inferior to 20% are considered adequate for the empirical use of a certain antibiotic^30,31^. According to Rossi *et al.* ^32^, fosfomycin and nitrofurantoin are the first choices for uncomplicated cystitis, the most prevalent UTI. Fosfomycin showed a resistance rate within the recommended limit for Gram-negative species, whereas the latter has a resistance percentage exactly at such limit, i.e., 20%.

Fosfomycin, however, is little used in Brazil, thus it is rarely tested in antibiograms. Furthermore, some authors suggest avoiding its use in favor of other appropriate first-choice antibiotics to prevent increasing resistance rates. Also note that fosfomycin is not used for the treatment of pyelonephritis since it does not reach adequate levels in the renal tissue^30^.

Nitrofurantoin is an antibiotic often used for the empirical treatment of uncomplicated cystitis. Despite a resistance rate at the recommended limit (20.0%) in our global analysis, this antibiotic showed resistance percentages inferior to this value in the evaluation of women (19.4%), of the three most frequent species (18.2%), and of *E. coli* singly (10.4%). Nitrofurantoin good efficiency is related to its ability to interfere in different mechanisms of bacterial metabolism^1^. The species *K. pneumoniae*, however, has high resistance to this drug (84.0% in this study), and *P. mirabilis* is intrinsically resistant. Additionally, this antibiotic must not be used for the treatment of pyelonephritis for the same reasons as fosfomycin^1^.

Rossi *et al*.^32^ suggest amoxicillin+clavulanate and cephalosporins (such as cefuroxime) as second choices for uncomplicated cystitis. Except for ceftazidime, both showed resistance rates greater than 20% in our global analysis, with the sensibility to amoxicillin+clavulanate higher than that to cephalosporins. Besides that, the resistance rates to these antibiotics are greater than those of the first-choice drugs, and they may cause more side effects^30,33^.

Sulfamethoxazole+trimethoprim has been traditionally used in uncomplicated cystitis, and, in some countries, it still figures as first-line therapy along with fosfomycin and nitrofurantoin^6^. In Brazil, however, the low susceptibility – as herein detected – made this drug not be recommended^32^. This fact possibly reflects the intense and irrational use of this drug^34^. Likewise, fluoroquinolones are not recommended for uncomplicated cystitis^32^. Besides high bacterial resistance, these drugs are also associated with severe adverse effects^35,36^, and some authors suggest avoiding their use to maintain their efficiency in the treatment of complicated cystitis and pyelonephritis^33^.

Regarding the treatment of uncomplicated pyelonephritis, quinolones figure as the first choice^31,37^. However, if local resistance to these drugs is higher than 10% – as found in this study, the American College of Clinical Pharmacy suggests giving ceftriaxone 1 g IV once or a dose of an aminoglycoside^31^. Sulfamethoxazole+Trimethoprim is quite effective against this type of UTI, but it is no longer considered an ideal agent for the empirical treatment of pyelonephritis due to the increasing rates of resistance, as herein detected. This antibiotic may be used only if pathogen sensitivity is observed in the uroculture^1,31^. Besides such options, in general lines, a range of drugs may be used: third and fourth-generation cephalosporins, amoxicillin+clavulanate, and piperacillin+tazobactam^31^. The choice must be based on patient conditions, allergies, and adherence, as well as on drug availability and cost^30^.

Another important result was the increase in resistance rates to many antimicrobials in older patients. This finding is quite expected since this group is at higher risk of infections caused by more resistant pathogens. These risk factors, for instance, include the previous use of antibiotics, the occurrence of recurrent UTIs, previous hospital admissions, indwelling urethral catheter, and invasive urinary instrumentation^34,38,39^.

Finally, previous hospital admission is widely accepted as a risk factor for resistant pathogens^38,40^. In fact, patients who had at least one hospitalization episode in the last 60 days had infections by bacteria with resistance rates significantly higher than those found for the total Gram-negative fermenting analysis, whereas the resistance rates were closer to those found for the Gram-negative fermenting analysis in patients with hospitalizations in the period of 61-90 days prior to the UTI. Furthermore, the logistic regression corroborated this finding. The odds of having infection by multidrug-resistant bacteria were 1.5 times higher in patients with previous hospitalization episodes in the last 90 day. This information may help guide the treatment plan for patients who have previously been hospitalized. For instance, ciprofloxacin and amoxicillin+clavulanate might not be suitable for people who have previously been hospitalized, especially within the last 30 days.

A limitation of our study that can be pointed out is the lack of access to some information, for example, the use of indwelling urinary catheters, the presence of renal diseases, anatomical abnormalities or comorbidities in patients, which could have improved our analysis. Besides that, we provide one-time information on antimicrobial resistance pattern and temporal variations make periodic reviews primordial.

## CONCLUSION

In brief, the empirical treatment of community-acquired UTI in both sexes should target the enterobacteria, especially *E. coli, K. pneumoniae*, and *P. mirabilis*. However, highlighting some peculiarities is key: the high prevalence of *P. mirabilis* in men under 12 years of age, as well as the high prevalence of *P. aeruginosa* and *E. faecalis* in people over 65 years of age of both sexes. In the studied location, the best options for treating lower UTI were nitrofurantoin (despite the high resistance of *K. pneumoniae* and the intrinsic resistance of *P. mirabillis*) and fosfomycin (which might be avoided to prevent increasing resistance rates), both antibiotics with a resistance rates inferior to 20%. If these options are not available, amoxicillin+clavulanate and cephalosporins, especially third generation agents, may be considered as second-line agents. The best options for treating pyelonephritis were aminoglycosides and carbapenems, followed by amoxicillin+clavulanate and cephalosporins. Sulfamethoxazole+trimethoprim and quinolones, traditionally prescribed for UTI, showed a high resistance rate and should be avoided.

The increase in resistance to the most commonly used antimicrobials is a serious global concern that is intensified by inadequate empirical therapy^40^. In view of that, we reinforce that treatment planning must consider local patterns of uropathogen prevalence and antimicrobial resistance rates, as well as other aspects such as gender, age, and previous hospitalization, to promote a rational use of antibiotics and avoid therapeutic failures. This study offers local basis for prescribing empirical antibiotics to the treatment of community-acquired UTI and these results can be used as reference by institutions with the same organization profile and without the resources to conduct a similar analysis.
